# In Silico Establishment and Validation of Novel Lipid Metabolism-Related Gene Signature in Bladder Cancer

**DOI:** 10.1155/2022/3170950

**Published:** 2022-04-18

**Authors:** Xianchao Sun, Ying Zhang, Yilai Chen, Shiyong Xin, Liang Jin, Xiang Liu, Zhen Zhou, Jiaxin Zhang, Wangli Mei, Bihui Zhang, Xudong Yao, Guosheng Yang, Lin Ye

**Affiliations:** ^1^Department of Urology, Shanghai East Hospital, School of Medicine, Tongji University, Shanghai 200120, China; ^2^Department of Urology, The Second Affiliated Hospital of Anhui Medical University, Hefei 230032, China; ^3^Department of Urology, Karamay People's Hospital, Xinjiang 834000, China; ^4^Department of Urology, Shanghai Tenth People's Hospital, School of Medicine, Tongji University, Shanghai 200072, China

## Abstract

**Background:**

Aberrant lipid metabolism is an alteration common to many types of cancer. Dysregulation of lipid metabolism is considered a major risk factor for bladder cancer. Accordingly, we focused on genes related to lipid metabolism and screened novel markers for predicting the prognosis of bladder cancer.

**Methods:**

RNA-seq data for bladder cancer were obtained from The Cancer Genome Atlas (TCGA) and Gene Expression Omnibus (GEO) databases. The nonnegative matrix factorization (NMF) algorithm was used to classify the molecular subtypes. Weighted correlation network analysis (WGCNA) was applied to identify coexpressed genes, and least absolute shrinkage and selection operator (LASSO) multivariate Cox analysis was used to construct a prognostic risk model. External validation data and *in vitro* experiments were used to verify the results from in silico analysis.

**Results:**

Bladder cancer samples were grouped into two clusters based on the NMF algorithm. A total of 1467 genes involved in coexpression modules were identified in WGCNA. We finally established a 5-gene signature (TM4SF1, KCNK5, FASN, IMPDH1, and KCNJ15) that exhibited good stability across different datasets and was also an independent risk factor for prognosis. Furthermore, the predictive efficacy of our model was generally higher than the predictive efficacy of other published models. Distinct risk groups of patients also showed significantly different immune infiltration cell patterns and associations with clinical variables. Moreover, the 5 signature genes were verified in clinical samples by quantitative real-time polymerase chain reaction (qRT-PCR) and immunohistochemistry, which were in agreement with the in silico analysis. For *in vitro* experiments, knockdown of IMPDH1 markedly inhibited cell proliferation in bladder cancer.

**Conclusion:**

We established a 5-gene prognosis signature based on lipid metabolism in bladder cancer, which could be an effective prognostic indicator.

## 1. Introduction

As one of the most common genitourinary malignancies, bladder cancer (BC) has become a global health problem. The development and progression of BC are a multistage sophisticated process that includes genetic characteristics and environmental factors [1]. Epidemiological studies have demonstrated that modification in dietary intake is associated with the risk of BC [2, 3]. A positive association between red and processed meat and the risk of BC has been reported [4]. Lipids are a ubiquitous class of structurally complex molecules composed of different fatty acids involved in various biological processes. Lipids play an integral role in homeostasis, cell membrane structure, and cell signaling [5]. Abnormal synthesis, degradation, digestion, and absorption of lipid substances in lipid metabolism contribute to excessive lipids in various tissues as well as tumorigenesis [6, 7].

Metabolic reprogramming is an important hallmark of cancer cells. Increased nutrient requirements are necessary to achieve rapid tumor growth and satisfy the metabolic needs of a tumor, and tumor cells have been observed to undergo metabolic reprogramming [8]. Tumor cells will choose the appropriate metabolic reprogramming method for metabolism so that they can better adapt to changing conditions. In tumorigenesis, fat is an important source of energy. In particular, researchers have found that lipid metabolic reprogramming exists in a variety of tumors, providing energy storage and intermediates of various metabolic activities for tumor proliferation, metastasis, and progression and even serving as a major intracellular metabolic type for cellular energy supply [9].

Recently, alterations in lipid metabolism have been recognized as a sign of many malignancies. Cheng et al. investigated whether fatty acid metabolism was activated in BC tissues, and inhibition of fatty acid oxidation showed a great impact on BC [10]. Previous studies have shown that general patterns and mechanisms of lipid metabolism participate in the development of BC, particularly blocking fatty acid synthesis to suppress the malignant phenotype of BC [11]. Furthermore, lipids mediate intercellular communication in the tumor-immune microenvironment. Lipid metabolism promotes the generation of M2-like tumor-associated macrophages (TAMs) and is crucial for TAM activity [12]. In tumor progression, cancer-stroma interactions are exacerbated, and fatty acids secreted in the microenvironment can influence the function and phenotype of infiltrating immune cells [13].

In this study, the expression of genes related to lipid metabolism in BC was investigated to screen hub genes that could predict patient prognosis. We developed and verified a 5-gene prognostic signature to effectively predict BC patient prognosis, as well as the relationship with immune infiltration cell patterns. This prognostic signature has the potential for clinical application in BC.

## 2. Methods

### 2.1. Data Preparation

Human lipid metabolism pathways were obtained from the Molecular Signature Database (MSigDB) [14], and 776 genes involved in lipid metabolism (Supplementary Table S1) were collated from 6 lipid metabolism pathways (Table 1). RNA-seq data were obtained from TCGA database for 433 BC samples, including 414 cancer samples and 19 normal samples, and corresponding clinicopathological information. RNA-seq data of 165 BC samples (GSE13507) and corresponding clinical information were downloaded from the GEO database.

### 2.2. Identification of Molecular Subtypes

First, we extracted 776 genes related to lipid metabolism from TCGA dataset; 24 genes were not identified, and 752 genes were included in subsequent analyses. Then, genes with significant differential expression were selected, and BC samples were clustered using a nonnegative matrix factorization (NMF) clustering algorithm with 50 iterations of the standard “brunet” [15]. The number of clusters *k* was set from 2 to 10, and the average contour width of the common member matrix was determined through the *R* package “NMF.” The optimal number of clusters was determined according to cophenetic, dispersion, and silhouette index.

### 2.3. Weighted Correlation Network Analysis (WGCNA)

The WGCNA algorithm was applied to screen coexpressed coding genes and modules according to the expression profile of protein-coding genes [16]. The soft threshold for network construction was determined by the criterion of approximate scale-free topology. After transformation of an adjacency matrix into a topological overlap matrix (TOM), genes were clustered with average linkage hierarchical clustering.

### 2.4. Gene Set Enrichment Analysis (GSEA)

To investigate the molecular mechanism, GSEA was applied [17]. The gene sets “c5.go.v7.4.symbols” and “c2.cp.kegg.v7.4.symbols” were obtained from the MSigDB database. An adjusted *p* value less than 0.05 was considered statistically significant. The *R* package “clusterProfiler” was used to carry out enrichment analyses.

### 2.5. Comprehensive Analysis of Immune Characteristics and Gene Mutations in Different Risk Groups

Kaplan-Meier survival curves were applied to compare prognoses between the two risk subgroups. To further investigate the gene mutations between different risk subgroups, we obtained information on genetic alterations from the cBioPortal database, and different gene mutations in the two risk subgroups were analyzed using the “Maftools” *R* package. To determine the immune profile of BC samples, their expression data were imported into CIBERSORT and iterated 1000 times to estimate the relative proportions of 22 types of immune cells. We then compared the relative proportions of immune cells and clinicopathological factors between different risk subgroups, and the results are presented as a landscape map.

### 2.6. Construction of the Nomogram

A nomogram is a visual model used to evaluate the prognosis of cancer. We therefore constructed a nomogram to predict the prognosis of patients with BC. Moreover, a calibration plot was constructed to estimate the accuracy and consistency of the prognostic model.

### 2.7. Analysis of DCA

Decision curve analysis (DCA) represents a novel method for evaluating clinical usefulness. DCA can determine the clinical utility based on the predictive nomogram, and the best model has a higher net benefit than the others.

### 2.8. Clinical Patients and Bladder Specimens

Fifty paired normal and tumor tissues were collected from BC patients who underwent radical cystectomy at Shanghai Tenth People's Hospital (Shanghai, China). Patients had diagnostic criteria in accordance with the World Health Organization (WHO) classification and did not receive any preoperative treatment. All patients provided informed consent prior to inclusion in the study, and ethical approval was obtained from the Ethics Committee of Shanghai Tenth People's Hospital. Detailed information is shown in Supplementary Table S2.

### 2.9. Cell Culture and Transfection

The immortalized human normal bladder epithelial cell line (SV-HUC-1) and bladder cancer T24, 5637, and UMUC3 cell lines were obtained from the Chinese Academy of Sciences (Shanghai, China). The UMUC3, T24, and 5637 cell lines were cultured in Roswell Park Memorial Institute- (RPMI-) 1640 medium (Gibco, USA), and the SV-HUC-1 cell line was maintained in F12K medium (Gibco). All cells were grown at 37°C in 5% CO_2_. The IMPDH1 siRNA (ATGGCTCTGATGGGAGGTATT), FASN siRNA (TGGCAAATTCGACCTTTCTCAGA), and TM4SF1siRNA (CGGCTAATATTTTGCTTTACTTT) were synthesized by Sangon Biotech (Shanghai, China). Lipofectamine 3000 (Invitrogen, USA) was used as a transfection reagent. T24 and UMUC3 cell lines were transfected with siRNA based on the protocol.

### 2.10. RNA Extraction and Quantitative Real-Time Polymerase Chain Reaction (qRT-PCR)

For the qRT-PCR assay, TRIzol (Invitrogen, USA) was used to extract the total RNA. Next, SYBR-Green Mix (Vazyme, Nanjing, China) with different primers (Sangon Biotech, China) was used according to the manufacturer's protocol. GAPDH was used as an internal control. Fold changes were calculated by the 2^-∆∆Ct^ method. Primer information is shown in Supplementary Table S3.

### 2.11. Cell Proliferation Assay

A total of 1 × 10^3^ cells were grown in each well of a 96-well plate. Cell viability was calculated with the cell counting kit-8 (CCK-8) system. The optical density (OD) value per well was measured at 450 nm (BioTek, USA).

### 2.12. Cell Colony Formation Assay

A total of 500 cells were grown in each well of 6-well plates for approximately 2 weeks until colony formation was evident. Then, the cells were fixed, stained, and photographed.

### 2.13. Western Blot

Western blot was carried out based on standard protocol. Briefly, cells were washed with chilled PBS and lysed with lysis solution. Total protein extracts were separated on a 10% SDS-PAGE gel and transferred onto PVDF membrane. After blocking with 5% nonfat dry milk, the PVDF membrane was incubated with primary antibody overnight at 4°C.

### 2.14. Immunohistochemistry (IHC)

Tissue samples were fixed and cut into 4 *μ*m slices. During dewaxing, rehydration, and antigen retrieval, sections were incubated with primary antibodies against TM4SF1, KCNK5, FASN, IMPDH1, and KCNJ15 (Thermo Fisher Scientific). Images were obtained with a microscope.

### 2.15. Statistical Analysis

Bioinformatics analyses were conducted using *R* version 4.1.1. GraphPad Prism 8 (GraphPad Software, Inc.) was used for statistical analysis. Student's *t* test or the Wilcoxon test was used to compare continuous data. The chi-square test and Fisher test were used to compare clinical and pathological parameters. Survival rates were assessed using Kaplan-Meier (K-M) curves and the log-rank test, and univariate and multivariate Cox regression was used to analyze the independent parameters associated with the overall survival. Pearson coefficient of correlation was calculated to measure the correlation between two variables. LASSO regression was carried out by using the “glmnet” *R* package to build a prognostic model. *R* software package “WGCNA” was used to construct a weighted coexpressed network. The correction between the risk signature and immune cells was analyzed by Spearman correlation analysis. The results of multivariate Cox regression analysis were visualized by the nomogram. *C*-index, time-dependent ROC curves, and calibration curves were used to evaluate the nomogram. All statistical *p* values were two-sided, and *p* < 0.05 was considered statistically significant.

## 3. Results

### 3.1. Molecular Subtypes Were Classified by the NMF Algorithm

**Figure 1:**
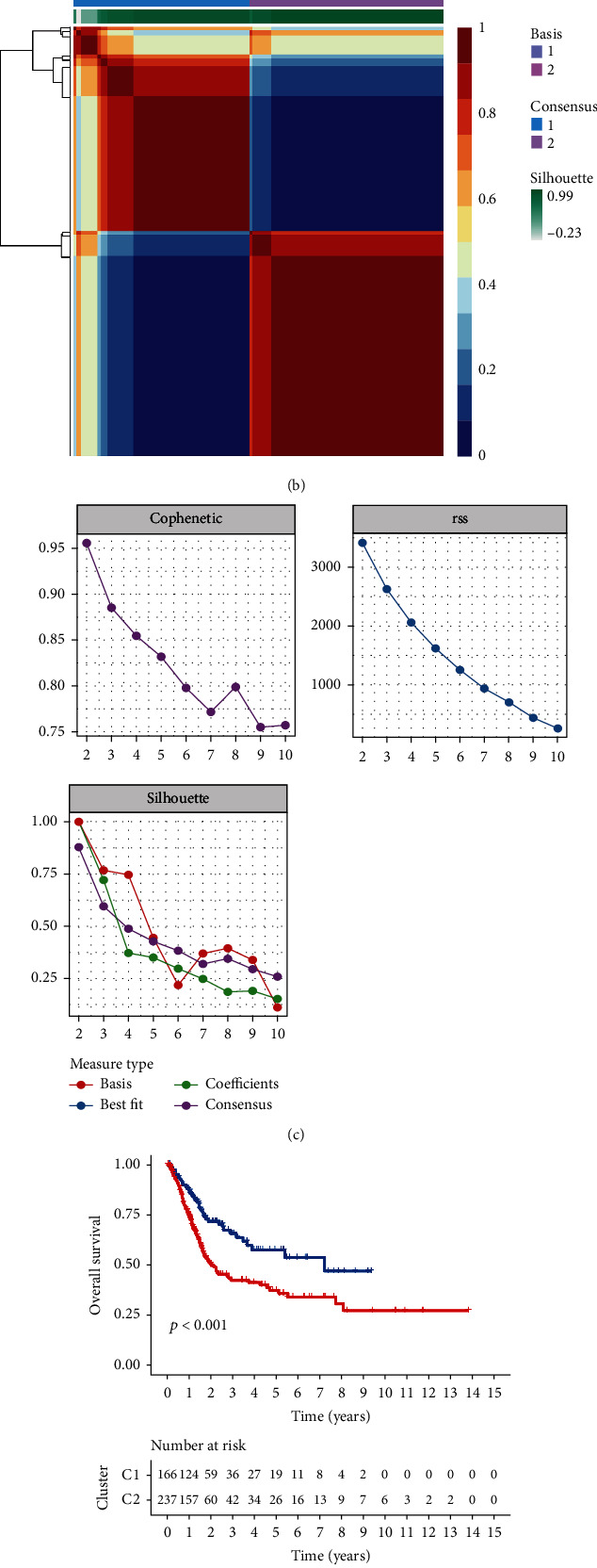
Cluster subtypes classified by the NMF algorithm. (a) Heatmap showing the differentially expressed lipid metabolism-related genes in TCGA. Red: upregulation; blue: downregulation. (b) NMF clustering consensus map. (c) NMF distributions when rank = 2-10. (d) Overall survival analysis of the two molecular subtypes. (e) Progression-free survival analysis of the two molecular subtypes.

Figure S1 presents the flow diagram of the current study. First, we selected six gene sets associated with lipid metabolism from MSigDB. To investigate gene expression in BC, RNA-seq data from TCGA bladder cancer cohort (TCGA-BLCA) were downloaded. The “limma” *R* package was used to screen the genes with differential expression. A total of 76 differentially expressed lipid metabolism-related genes on BC were obtained (*p* < 0.05, Figure 1(a)). Next, BC samples were clustered using the NMF method with 50 iterations by the “brunet” selection criterion. The optimal number of clusters chosen based on cophenetic, dispersion, and silhouette is *k* = 2. (Figures 1(b) and 1(c)). The prognostic relationship between Cluster 1 (C1) and Cluster 2 (C2) with overall survival (OS) (Figure 1(d), log rank *p* < 0.001) and progression-free survival (Figure 1(e), log rank *p* = 0.006) showed that subgroup C1 exhibited better prognosis than subgroup C2.

### 3.2. Identification of Functional Modules by WGCNA

**Figure 2:**
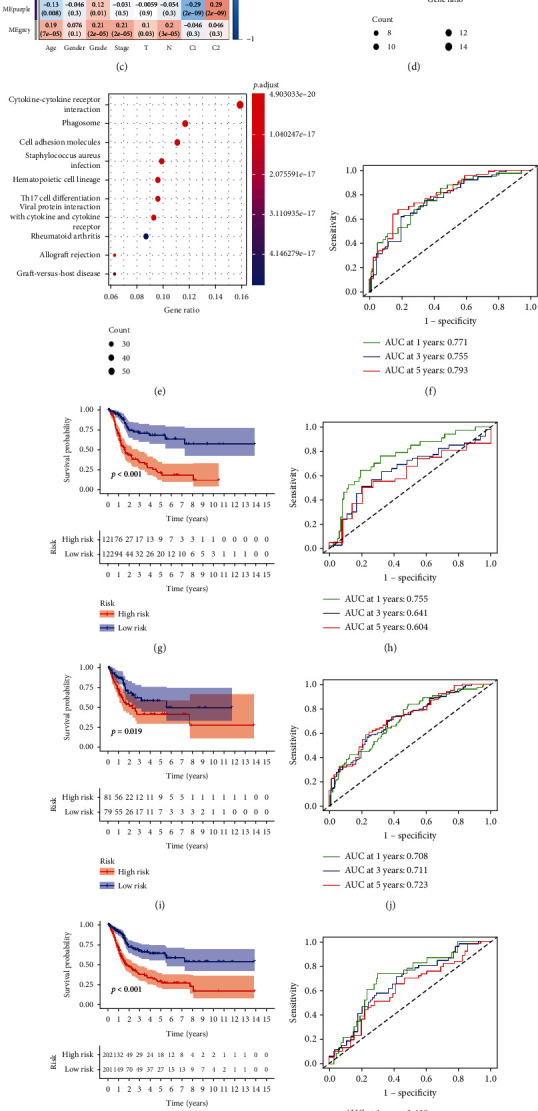
Identification of modules and construction of the model. (a) Cluster analysis and soft-thresholding powers. (b) WGCNA module colors. (c) Correlations of thirteen modules with clinical variables and clusters. (d, e) KEGG enrichment in the turquoise and brown modules. (f) ROC curve of the model in TCGA training cohort. (g) Survival curves of the groups in TCGA training cohort. (h) ROC curve of the model in TCGA testing cohort. (i) Survival curves of the groups in TCGA testing cohort. (j) ROC curve of the model in the entire TCGA cohort. (k) Survival curves of the groups in the entire TCGA cohort. (l) ROC curve of the model in the GSE13507 cohort. (m) Survival curves of the groups in the GSE13507 cohort.

The WGCNA algorithm was used to identify coexpressed coding genes and modules according to the expression profile of protein-coding genes. Hierarchical clustering analysis showed no outlier samples, and a soft threshold of 3 was chosen (Figure 2(a)). Genes were clustered using average linkage hierarchy clustering to obtain 13 modules with height = 0.25, deepSplit = 2, and minModuleSize = 30 (Figures 2(b) and 2(c)). Correlations of the modules with gender, age, clinical stage, N stage, T stage, C1, and C2 were further analyzed, in which modules significantly correlated with C1 and C2 were turquoise and brown modules. The turquoise module contained 907 genes, and the brown module contained 560 genes (Supplementary Table S4). These 1467 genes were used to establish the prognostic risk model. Analysis of Gene Ontology (GO) and Kyoto Encyclopedia of Genes and Genomes (KEGG) pathway enrichment in the two module genes was performed using the *R* package “cluster profiler.” The top 10 GO terms (Figures S2A and S2B) and KEGG pathways (Figures 2(d) and 2(e)) in the turquoise and brown modules were obtained.

### 3.3. Establishment of the Prognostic Risk Model

BC samples were divided randomly into a training cohort and testing cohort in a 6 : 4 ratio. The detailed information of the patient cohorts is summarized in Table 2. Univariate Cox proportional hazard analysis was performed to screen prognosis-related significant genes in the training set. Based on a threshold *p* value less than 0.01, 46 genes were identified with significant prognostic differences (Supplementary Table S5). To further narrow the range of genes and construct a highly accurate prognostic model, LASSO Cox regression and multivariate Cox analysis were used to select the hub genes (Figures S3A and S3B). Combining the analysis, 5 target genes were selected. The 5-gene signature formula was as follows: risk score = expression level of TM4SF1∗(0.197) + expression level of KCNK5∗(−0.373) + expression level of FASN∗(0.408) + expression level of IMPDH1∗(0.342) + expression level of KCNJ15∗(−0.171). Then, we analyzed the predictive classification efficiencies of the model, and the area under the curve (AUC) values for 1, 3, and 5 years were 0.771, 0.755, and 0.793, respectively, indicating good predictive performance (Figure 2(f)). Moreover, we divided the risk score into two risk subgroups, plotted the K-M curve as shown in Figure 2(g), and found a significant difference between them (*p* < 0.001). To further determine the robustness of this model, we used the same coefficients in the internal testing cohort, all TCGA cohorts, and the independent validation cohort GSE13507. In the testing cohort, the AUC values for 1, 3, and 5 years were 0.755, 0.641, and 0.604, respectively (Figure 2(h)); in all TCGA cohorts, the AUC values for 1, 3, and 5 years were 0.708, 0.711, and 0.723, respectively (Figure 2(i)). In the GSE13507 cohort, the AUC values for 1, 3, and 5 years were 0.699, 0.666, and 0.614, respectively (Figure 2(l)). Similar results were obtained in these cohorts, and significant differences were observed between the two risk subgroups (Figures 2(i), 2(k), and 2(m)). These results showed that the constructed risk signature has good robustness and could be used to predict the prognostic risk of BC patients in different cohorts.

### 3.4. Molecular Characteristics between Different Risk Subgroups

**Figure 3:**
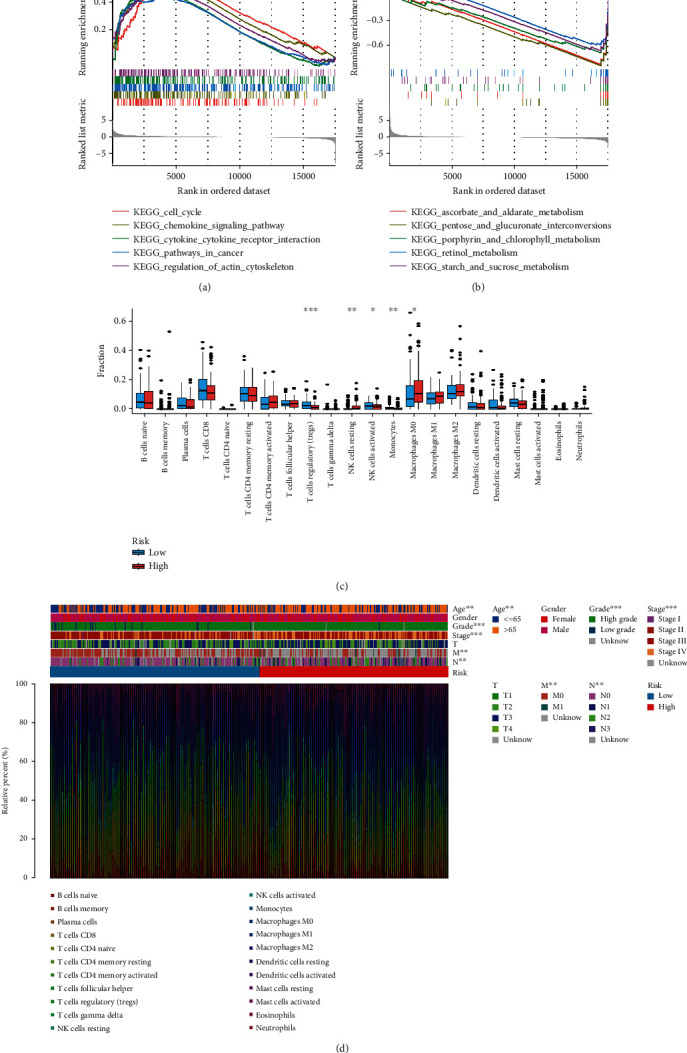
Molecular characteristics of the two risk subgroups and correlation between the risk model and immunity. GSEA for high-risk patients (a) and low-risk patients (b). (c) The relative proportions of 22 kinds of immune cells in the two risk subgroups. (d) Clinicopathological characteristics of two risk subgroups with the immune landscape.

Mutation analysis in TCGA-BLCA samples revealed that 3%, 2.4%, 6%, 3%, and 2.2% of patients had mutations in TM4SF1, KCNK5, FASN, KCNJ15, and IMPDH1, respectively (Figure S4A). Missense variations, nonsense variations, and frameshift deletions were the common mutation types between the subgroups. The mutation rates of TTN, TP53, ARID1A, MUC16, KDM6A, and KMT2D were more than 20% in the two subgroups (Figures S4B and S4C). GSEA was used to identify the gene sets enriched in different risk subgroups. Gene sets from samples with high-risk score samples were enriched in pathways related to the cell cycle, chemokine signaling pathway, cytokine-cytokine receptor, pathways in cancer, and regulation of actin cytoskeleton, while the gene sets of low-risk score samples were enriched in metabolism pathways (Figures 3(a) and 3(b)).

### 3.5. Prognostic Analysis of Risk and Clinical Characteristics

We further compared the risk score between patients with different clinical characteristics. Correlation analysis of the risk score and clinical characteristics such as age, pathological grade, clinical stage, N stage, T stage, and M stage revealed a statistically significant association (Figure S5). We further performed survival analysis of the clinical subgroup with the risk score. As shown in Figure S6, the 5-gene signature can clearly distinguish patients by age, gender, T stage, and N stage. These results suggested that our risk model retained strong power in predicting different clinical variables. Furthermore, univariate and multivariate Cox regression analyses were used to investigate the independence of the risk (Table 3). The proportional hazards assumption was tested using the Schoenfeld residual test (Figure S7). Univariate Cox regression analysis showed that age, stage, T stage, N stage, and risk were closely related to survival. Based on multivariate analysis, only age (HR = 1.042, 95%CI = [1.023 − 1.062], *p* = 1.48*E* − 05), T stage (HR = 1.395, 95%CI = [1.021 − 1.908], *p* = 0.0365), and risk score (HR = 1.337, 95%CI = [1.195 − 1.496], *p* = 3.66*E* − 07) were significantly associated with survival. This finding indicated that this 5-gene signature was an independent risk factor for predicting prognosis.

### 3.6. Correlation between the Risk Model and Immunity

To explore the indicative roles of this risk model on the tumor microenvironment (TME), CIBERSORT was adopted to evaluate the relative proportion of 22 kinds of immune cells [18]. The results showed that the high-risk subgroup was significantly associated with natural killer (NK) resting cells and M0 macrophages, while the low-risk subgroup was significantly associated with regulatory T cells (Tregs), NK activated cell, and monocytes (Figure 3(c)). The correlations between the clinicopathological characteristics of different risk subgroups and the immune landscape are displayed in Figure 3(d). The results showed that our risk model could potentially reflect the status of the TME.

Furthermore, we explored whether the risk model had the potential to predict treatment response to immune checkpoint inhibitors (ICIs). We found that the expression of PD-1, PD-L1, LAG3, and CTLA4 was markedly higher in the high-risk subgroup, indicating a positive correlation with risk (Figures S8B–S8E). Moreover, the enrichment scores of immune-related pathways were quantified. Interestingly, most of the functions associated with antigen presentation, such as T cell costimulation and MHC and APC coinhibition, showed a bias toward the high-risk group (Figure S8A).

### 3.7. Comparison of the Risk Model with Other Signatures

**Figure 4:**
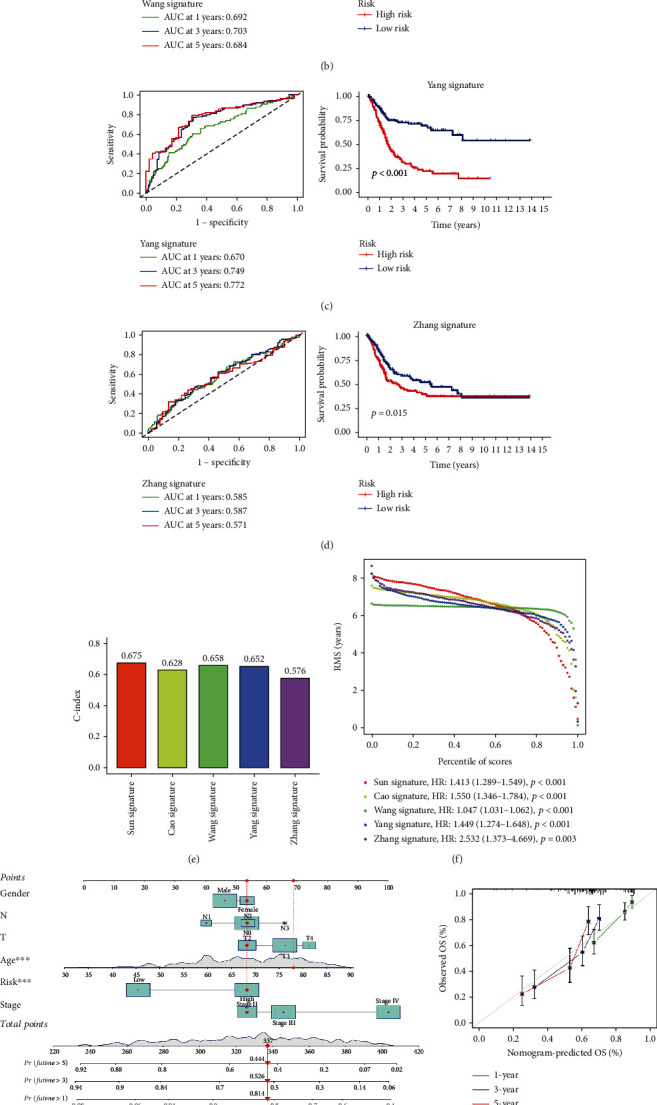
Comparison with other signatures and construction of the nomogram. (a) ROC and survival curves of the Cao signature. (b) ROC and survival curves of the Wang signature. (c) ROC and survival curves of the Yang signature. (d) ROC and survival curves of the Zhang signature. (e) *C*-index between the five signatures. (f) RMS result of the five signatures. (g) A nomogram to predict overall survival. (h) Calibration plots for prediction. (i) DCA for the nomogram, risk, and clinical variables.

Four prognostic risk signatures, the 4-gene, 5-gene, 7-gene, and 9-gene signatures, were chosen for comparison with this risk signature. We also used the same method to calculate every risk score in TCGA dataset according to the corresponding genes in the four risk signatures. Survival differences in the low- and high-risk subgroups were also detected with these risk signatures (Figures 4(a)–4(d), log rank *p* < 0.05). To further validate prognostic prediction performance among them, the *R* package “rms” was used to calculate the concordance index (*C*-index). The results showed that our model had the highest *C*-index, highlighting a better performance (Figures 4(e) and 4(f)).

### 3.8. Construction of the Nomogram with Risk Score

To evaluate the potential clinical practicality of the prognostic model, we combined the clinicopathological features and risk to construct a nomogram. As shown in Figure 4(g), a prognostic nomogram with risk score and clinical variables was constructed. The 1-, 3-, and 5-year calibration plots demonstrated the performance of the nomogram (Figure 4(h)). Finally, DCA indicated that the nomogram had a higher overall net benefit for clinical utility (Figure 4(i)).

### 3.9. Clinical and In Vitro Validation of 5 Gene Expressions

**Figure 5:**
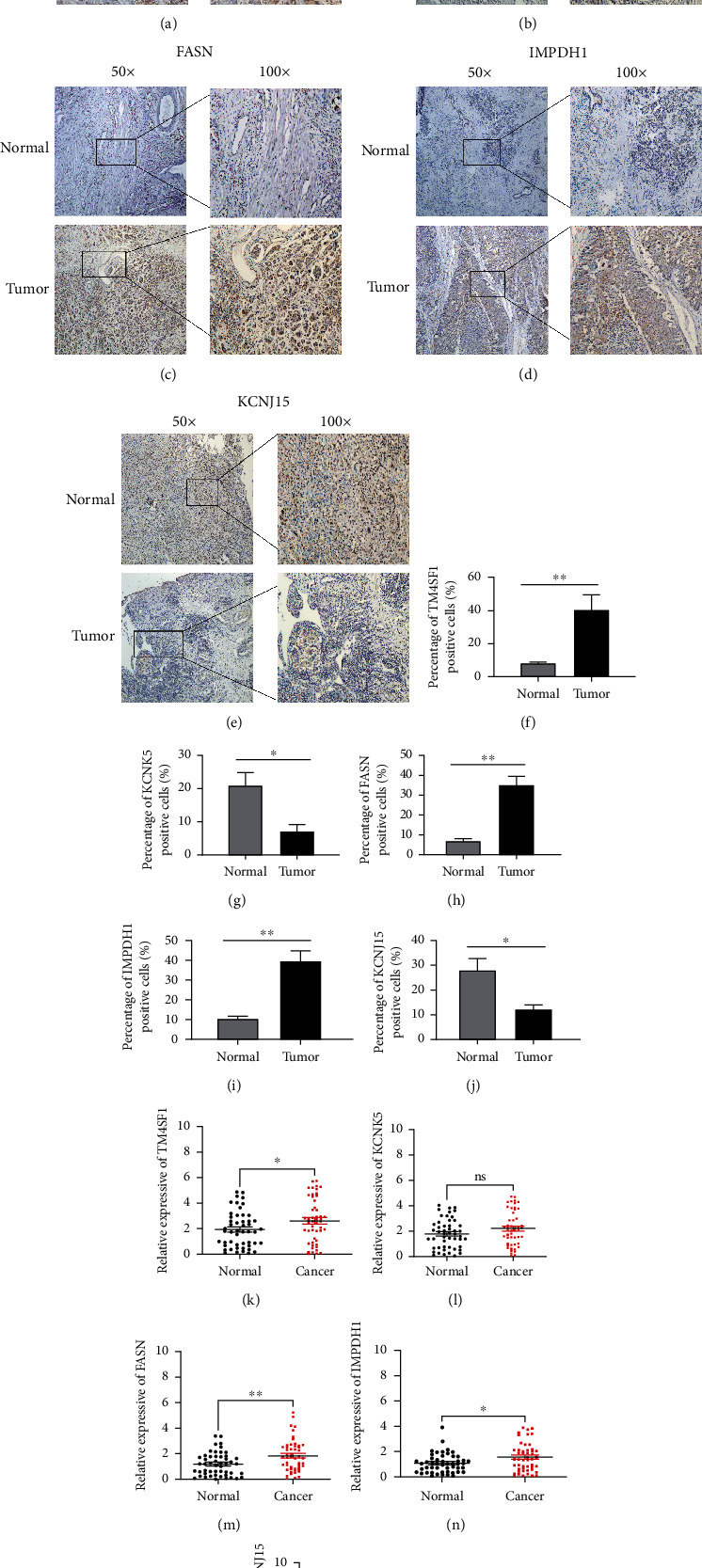
Immunohistochemistry and qRT-PCR results of the 5 genes in tissue samples. The protein expression of TM4SF1 (a), KCNK5 (b), FASN (c), IMPDH1 (d), and KCNJ15 (e) in clinical samples. (f–j) Percentage of positive cells of the 5 genes. (k–o) qRT-PCR analysis of TM4SF1, FASN, IMPDH1, KCNJ15, and KCNK5 mRNA levels in tissue samples. ^∗^*p* < 0.05, ^∗∗^*p* < 0.01, ^∗∗∗^*p* < 0.001, and ns *p* > 0.05.

**Figure 6:**
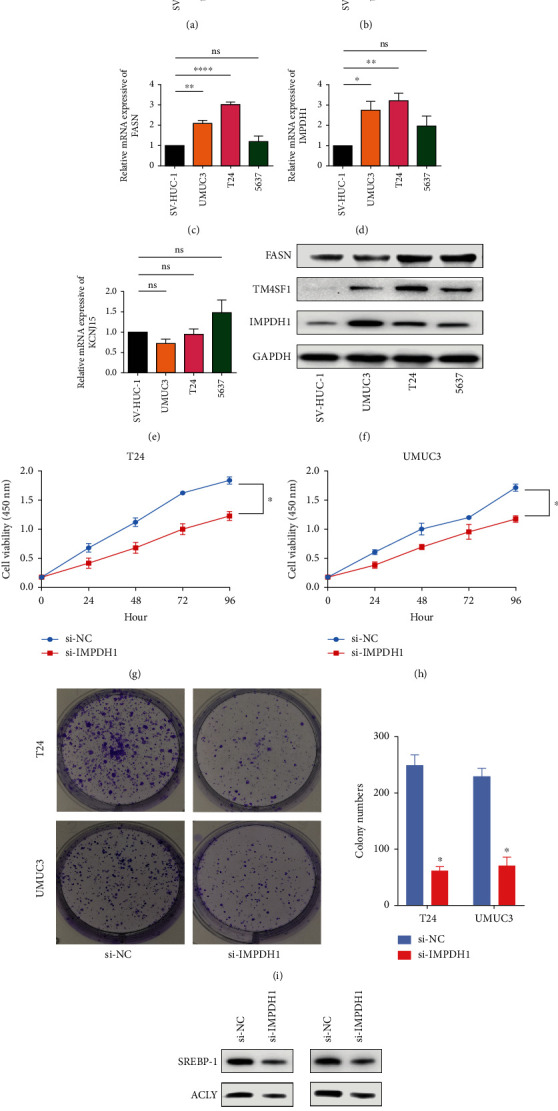
*In vitro* validation of the expression of 5 genes. (a–e) qRT-PCR analysis of TM4SF1, FASN, IMPDH1, KCNJ15, and KCNK5 mRNA levels in cell lines. (f) The protein expression levels of the identified genes (TM4SF1, FASN, and IMPDH1) in cell lines by western blot. (g–i) CCK-8 and colony formation assays showed that silencing IMPDH1 suppressed cell proliferation. (j) Downregulation of IMPDH1 decreased the protein levels of SREBP-1 and ACLY in T24 and UMUC3 cells. ^∗^*p* < 0.05, ^∗∗^*p* < 0.01, ^∗∗∗^*p* < 0.001, and ns *p* > 0.05.

To further confirm the above results, 50 cases of BC tissue specimens were included. Immunohistochemistry results showed that TM4SF1, FASN, and IMPDH1 were significantly highly expressed in cancer tissues, and KCNK5 and KCNJ15 were highly expressed in normal tissues (Figures 5(a)–5(j)). The results were basically consistent with the in silico analysis. The findings also showed that the mRNA expression levels of TM4SF1, FASN, and IMPDH1 were higher in cancer tissues (Figures 5(k), 5(m), and 5(n)), whereas the mRNA expression of KCNJ15 was higher in normal tissues (Figure 5(o)). Moreover, the current study findings showed that the levels of TM4SF1, FASN, and IMPDH1 were significantly increased in BC cell lines (UMUC-3 and T24) compared with SV-HUC-1 (Figures 6(a)–6(f)). The qRT-PCR and western blotting validation findings confirmed the important roles of the signature genes in BC. Next, we analyzed the potential function of IMPDH1 in BC. Silencing IMPDH1 inhibited the proliferation and colony formation of bladder cancer cells *in vitro* (Figures 6(g)–6(i)). The results indicated that IMPDH1 may act as an oncogene in BC. Similar results were also observed after silencing FASN or TM4SF1 in bladder cancer cells (Figure S9). We further investigated the influence of IMPDH1 on lipid metabolism-related pathways; the expressions of two key enzymes in the fatty acid synthesis pathway were measured in IMPDH1 knockdown and control bladder cancer cells. As shown in Figure 6(j), downregulation of IMPDH1 decreased the protein levels of SREBP-1 and ACLY in T24 and UMUC3 cells. Thus, we suggest that IMPDH1 plays an important role in regulating lipid metabolism on BC.

## 4. Discussion

BC is a common malignant tumor worldwide and has high rates of progression and recurrence. Despite advances in surgery and comprehensive treatment regimens, effective targeted therapies and prognostic markers are still lacking in patients with BC [19]. In the process of transforming normal cells into malignant cells, one of the key steps is metabolic reprogramming, including glycolysis and glutamine and fatty acid metabolism [20]. There is growing evidence that certain specific changes in lipid metabolism have an impact on the synthesis and degradation of tumor cell membranes, thus maintaining the energy balance in the body [21, 22].

In the present study, we identified two subtypes of BC by using the NMF algorithm based on genes related to lipid metabolism. Next, WGCNA was applied to screen modules of coexpressed genes, and 1467 genes were obtained. Then, LASSO and multivariate Cox analyses were performed to construct a 5-gene prognostic risk model. Our findings showed that this risk model had stable performance in the prognosis of BC patients and was implicated in clinical characteristics and immune infiltration cells.

This risk signature was constructed with TM4SF1, KCNK5, FASN, IMPDH1, and KCNJ15. According to our study results, TM4SF1, FASN, and IMPDH1 were significantly and highly expressed in BC tissues, while KCNK5 and KCNJ15 were poorly expressed. In accordance with the corresponding coefficients of these five genes, the risk score was calculated, and samples were grouped with different risks. Survival analysis exhibited significant discrepancies between different risk subgroups. We also confirmed that the risk score was an independent prognostic factor for survival. Stratified survival analysis also indicated the good feasibility of this model. Furthermore, CIBERSORT showed that patients in the high-risk subgroup had higher proportions of M0 macrophages and NK resting cells, while Tregs and NK-activated cells were upregulated in the low-risk group, indicating a differential infiltration pattern between the subgroups [23]. GSEA indicated that the cell cycle, regulation of actin cytoskeleton, pathways in cancer, cytokine-cytokine receptor interaction, and chemokine signaling pathway increased with risk score, suggesting that risk was closely related to tumor development and immune activity [24, 25]. Immune checkpoints could be regarded as potential therapeutic targets of tumor treatment, and the inhibitors have exhibited superior anticancer efficacy [26]. We found that the high-risk subgroup was associated with immune checkpoints such as PD-1, PD-L1, LAG3, and CTLA-4, implying that patients with different risks respond differently to immunotherapy.

TM4SF1 acts as an integral membrane glycoprotein that transmits signals from the extracellular space to the cytoplasm. Previous studies have shown that TM4SF1 is overexpressed in various cancers and is strongly upregulated in BC tissues [27]. FASN is a key enzyme in fatty acid synthesis. FASN levels have been reported to be closely associated with cell proliferation and apoptosis. Research on the relationship between FASN and BC has proven that FASN is associated with the progression of BC [28]. IMPDH1 is a key regulator of GTP synthesis and is required for signal transduction. IMPDH1 has been indicated to be positively associated with renal cancer metastasis [29]. KCNK5 is a member of the KCNK family, which can regulate membrane potential and excitability. The expression of KCNK5 is associated with the maintenance of hearing [30]. KCNJ15 is a member of the inwardly rectifying potassium channel family. Liu et al. demonstrated that KCNJ15 inhibits renal cell proliferation [31].

Next, we further validated the expression of these five genes in tissue specimens and cell lines. The immunohistochemistry results suggested that the expression of TM4SF1, FASN, and IMPDH1 was significantly elevated in tumor tissue specimens, while the expression of KCNK5 and KCNJ15 was downregulated. The mRNA expression levels detected in the tissue specimens were also generally consistent with the above results. In an *in vitro* experiment, we demonstrated that TM4SF1, FASN, and IMPDH1 were elevated in some bladder cancer cell lines. These results are in accordance with the bioinformatics analysis. Finally, we focused on the function of IMPDH1 in BC and found that cell proliferation and colony formation were significantly inhibited after knocking down IMPDH1, implying that IMPDH1 has a tumor-promoting effect.

Previous studies have also developed cancer biomarkers associated with BC prognosis. Cao et al. constructed a 7-gene signature related to EMT for predicting the outcome of BC patients [32], while a 9-gene signature related to ferroptosis was established by Yang et al. [33]. Zhang et al. identified a glycolysis-based 4-mRNA signature to promote therapeutic options in BC [34]. Wang et al. used univariate and robust methods to establish a 5-gene risk model in BC [35]. To further examine the advantages of our signature, we compared these risk models simultaneously. The results demonstrated that our model had the highest *C*-index, exhibiting good application value compared with the others. Our risk signature obtained a more stable and effective prediction with fewer genes, which is better suited for clinical applications. Although the results were derived from multiomics data with a large sample, some limitations also need to be improved. Despite the rigorous bioinformatics analysis in the current study, further verification is still needed.

Aberrant activities of lipid metabolism have unique functional roles in cancer progression, and their imbalanced status has been a topic for screening therapeutic targets. Therefore, we attempted to fill the knowledge gap between lipid gene status and prognosis prediction in BC. Based on this study, we think these five genes may be implicated in lipid metabolism processes, and this risk signature may serve as a prognostic biomarker for BC treatment.

In conclusion, we established and validated a 5-gene signature associated with lipid metabolism, which was an independent prognostic factor in different datasets. The risk signature exhibited superior predictive performance compared to other published models. This 5-gene signature could be recognized as a prognostic marker to reflect the survival of BC.

## Figures and Tables

**Table 1:** Six pathways involved in lipid metabolism.

Pathway	Database	Gene count
Peroxisome proliferator-activated receptor alpha	Reactome	119
Metabolism of lipids	Reactome	738
Transcriptional regulation of white adipocyte differentiation	Reactome	84
Sphingolipid metabolism	Reactome	89
Glycerophospholipid metabolism	KEGG	77
Fatty acid metabolism	Reactome	177
	Total: 1284	
	Unique: 776	

**Table 2:** Clinical characteristics of bladder cancer patients from TCGA and GSE13507 datasets.

Clinical characteristics	TCGA cohort (*n* = 412)	TCGA train (*n* = 243)	TCGA test (*n* = 160)	GSE13507 cohort (*n* = 165)
Age at diagnosis (year)				
≤65	162	95	65	74
>65	250	148	95	91
Gender				
Male	304	177	121	135
Female	108	66	39	30
Grade				
Low grade	21	10	10	105
High grade	388	233	147	60
Stage				
I/II	133	77	53	135
III/IV	277	166	105	30

**Table 3:** Univariate and multivariate cox analysis.

Clinical variables	Univariable analysis			Multivariable analysis		
	HR	95% CI	*p* value	HR	95% CI	*p* value
Age	1.040	1.021-1.059	2.75*E*-05	1.042	1.023-1.062	1.48*E*-05
Gender	1.110	0.761-1.620	0.5860	1.192	0.812-1.750	0.3679
Stage	1.951	1.524-2.497	1.10*E*-07	1.279	0.827-1.978	0.2669
T	1.658	1.275-2.154	0.0001	1.395	1.021-1.908	0.0365
N	1.592	1.334-1.901	2.58*E*-07	1.214	0.893-1.650	0.2154
Risk score	1.371	1.238-1.517	1.24*E*-09	1.337	1.195-1.496	3.66*E*-07

## Data Availability

The authors declare that the data supporting the findings of the current study are provided in the article. Datasets analyzed for this work can be obtained from TCGA (https://portal.gdc.cancer.gov/), GEO (https://www.ncbi.nlm.nih.gov/geo/), MSigDB (https://www.gsea-msigdb.org/gsea/msigdb), cBioPortal (https://www.cbioportal.org/), and CIBERSORT (https://cibersort.stanford.edu/).

## Abbreviations

NMF: Nonnegative matrix factorization
WGCNA: Weighted correlation network analysis
BC: Bladder cancer
TCGA: The Cancer Genome Atlas
GEO: Gene Expression Omnibus
ROC: Receiver-operating characteristic
OS: Overall survival
LASSO: Least absolute shrinkage and selection operator
AUC: Areas under the curve
TME: Tumor microenvironment
DCA: Decision curve analysis
GSEA: Gene set enrichment analysis
GO: Gene Ontology
KEGG: Kyoto Encyclopedia of Genes and Genomes
ICIs: Immune checkpoint inhibitors
*C*-index: Concordance index
NC: Negative control.

## Supplementary Materials

Supplementary Materials Figure S1: flow diagram of the current study. Figure S2: GO analysis of coexpressed genes. GO enrichment of genes in the turquoise (A) and brown (B) modules. Figure S3: construction of the model. (A) LASSO coefficient profile plot. (B) The values of lambda in the model. Figure S4: gene mutations between the two risk subgroups. (A) Mutation analysis of the five genes. Gene mutation distributions in high-risk (B) and low-risk subgroups (C). Figure S5: correlation analysis of risk score with clinical characteristics in age (A), grade (B), clinical stage (C), M stage (D), N stage (E), and T stage (F). Figure S6: stratified survival analysis. (A) Survival analysis of two risk subgroups in age ≤ 65 years. (B) Survival analysis of two risk subgroups in age > 65. (C) Survival analysis of two risk subgroups in females. (D) Survival analysis of two risk subgroups in males. (E) Survival analysis of two risk subgroups in stages I + II. (F) Survival analysis of two risk subgroups in stages III + IV. (G) Survival analysis of two risk subgroups in T1 + T2. (H) Survival analysis of two risk subgroups in T3 + T4. (I) Survival analysis of two risk subgroups in N0. (J) Survival analysis of two risk subgroups in N1 + N2 + N3. Figure S7. The proportional hazards assumption was tested using the Schoenfeld residual test. Figure S8: correlation between the risk model and immune checkpoint inhibitors. (A) Immune-related pathways were quantified between the two risk subgroups. The relationship between risk score and PD-1 (B), PD-L1 (C), CTLA4 (D), and LAG3 (E). Figure S9: CCK-8 assay showed that silencing FASN (A, B) or TM4SF1 (C, D) suppressed cell proliferation in T24 and UMUC3 cell lines. Table S1: lipid metabolism-related genes. Table S2: clinical characteristics of 50 bladder cancer patients. Table S3: primer information. Table S4: 907 genes in turquoise module and 560 genes in brown module. Table S5: 46 genes with significant prognostic differences.
