# Supplementary material for: In Silico Establishment and Validation of Novel Lipid Metabolism-Related Gene Signature in Bladder Cancer
Source: Oxid Med Cell Longev. 2022 Apr 18;2022:3170950. doi: 10.1155/2022/3170950 (PMC9038413; doi:10.1155/2022/3170950)
Supplement: Supplementary Materials — Figure S1: flow diagram of the current study. Figure S2: GO analysis of coexpressed genes. GO enrichment of genes in the turquoise (A) and brown (B) modules. Figure S3: construction of the model. (A) LASSO coefficient profile plot. (B) The values of lambda in the model. Figure S4: gene mutations between the two risk subgroups. (A) Mutation analysis of the five genes. Gene mutation distributions in high-risk (B) and low-risk subgroups (C). Figure S5: correlation analysis of risk score with clinical characteristics in age (A), grade (B), clinical stage (C), M stage (D), N stage (E), and T stage (F). Figure S6: stratified survival analysis. (A) Survival analysis of two risk subgroups in age ≤ 65 years. (B) Survival analysis of two risk subgroups in age > 65. (C) Survival analysis of two risk subgroups in females. (D) Survival analysis of two risk subgroups in males. (E) Survival analysis of two risk subgroups in stages I + II. (F) Survival analysis of two risk subgroups in stages III + IV. (G) Survival analysis of two risk subgroups in T1 + T2. (H) Survival analysis of two risk subgroups in T3 + T4. (I) Survival analysis of two risk subgroups in N0. (J) Survival analysis of two risk subgroups in N1 + N2 + N3. Figure S7. The proportional hazards assumption was tested using the Schoenfeld residual test. Figure S8: correlation between the risk model and immune checkpoint inhibitors. (A) Immune-related pathways were quantified between the two risk subgroups. The relationship between risk score and PD-1 (B), PD-L1 (C), CTLA4 (D), and LAG3 (E). Figure S9: CCK-8 assay showed that silencing FASN (A, B) or TM4SF1 (C, D) suppressed cell proliferation in T24 and UMUC3 cell lines. Table S1: lipid metabolism-related genes. Table S2: clinical characteristics of 50 bladder cancer patients. Table S3: primer information. Table S4: 907 genes in turquoise module and 560 genes in brown module. Table S5: 46 genes with significant prognostic differences. [file 3170950.f1.zip › Table S3. Primer information.docx]

**Table S3: Primer information.**

Primer sequence.

| **Gene Name** | **Primer Sequence** |
| --- | --- |
| **GAPDH** | F: AATGGGCAGCCGTTAGGAAA  R: GCCCAATACGACCAAATCAGAG |
| **TM4SF1** | F: CCTCTTGGCTCTTGGTGGAA  R: TAGCAGTCATATTGCTGTTGGT |
| **KCNK5** | F: GAGAGGTGTGAGTCTGCGGAA  R: TTCCACCCCTCAGTCACCAT |
| **FASN** | F: GTCTTGAACTCCTTGGCGGA  R: AGGAAGATAGCCATGCCGAG |
| **IMPDH1** | F: CGACACCCCGTTCAGAACTAT  R: GCTGATCAGGTAGTCCGCC |
| **KCNJ15** | F: TCTTCAGTCTTTGCAGGCAGTA  R: GGGAACAAGCTGTCTGTGAGG |

F: Forward; R: Reverse.
