# Supplementary material for: In Silico Establishment and Validation of Novel Lipid Metabolism-Related Gene Signature in Bladder Cancer
Source: Oxid Med Cell Longev. 2022 Apr 18;2022:3170950. doi: 10.1155/2022/3170950 (PMC9038413; doi:10.1155/2022/3170950)
Supplement: Supplementary Materials — Figure S1: flow diagram of the current study. Figure S2: GO analysis of coexpressed genes. GO enrichment of genes in the turquoise (A) and brown (B) modules. Figure S3: construction of the model. (A) LASSO coefficient profile plot. (B) The values of lambda in the model. Figure S4: gene mutations between the two risk subgroups. (A) Mutation analysis of the five genes. Gene mutation distributions in high-risk (B) and low-risk subgroups (C). Figure S5: correlation analysis of risk score with clinical characteristics in age (A), grade (B), clinical stage (C), M stage (D), N stage (E), and T stage (F). Figure S6: stratified survival analysis. (A) Survival analysis of two risk subgroups in age ≤ 65 years. (B) Survival analysis of two risk subgroups in age > 65. (C) Survival analysis of two risk subgroups in females. (D) Survival analysis of two risk subgroups in males. (E) Survival analysis of two risk subgroups in stages I + II. (F) Survival analysis of two risk subgroups in stages III + IV. (G) Survival analysis of two risk subgroups in T1 + T2. (H) Survival analysis of two risk subgroups in T3 + T4. (I) Survival analysis of two risk subgroups in N0. (J) Survival analysis of two risk subgroups in N1 + N2 + N3. Figure S7. The proportional hazards assumption was tested using the Schoenfeld residual test. Figure S8: correlation between the risk model and immune checkpoint inhibitors. (A) Immune-related pathways were quantified between the two risk subgroups. The relationship between risk score and PD-1 (B), PD-L1 (C), CTLA4 (D), and LAG3 (E). Figure S9: CCK-8 assay showed that silencing FASN (A, B) or TM4SF1 (C, D) suppressed cell proliferation in T24 and UMUC3 cell lines. Table S1: lipid metabolism-related genes. Table S2: clinical characteristics of 50 bladder cancer patients. Table S3: primer information. Table S4: 907 genes in turquoise module and 560 genes in brown module. Table S5: 46 genes with significant prognostic differences. [file 3170950.f1.zip › Table S1. Lipid metabolism-related genes.doc]

**Table S1: Lipid metabolism-related genes.**

NFYA

CREBBP

ABCB4

MED24

NR1H4

ALAS1

NR1H3

MED17

THRAP3

MED29

AHRR

ME1

NFYC

RORA

SREBF1

FDFT1

SMARCD3

NCOA1

MED15

EP300

TRIB3

TBL1X

MED25

MED26

SULT2A1

NRF1

AHR

MED13

MED31

PPARGC1A

CPT1A

APOA5

CCNC

MED23

FAM120B

HMGCS1

HMGCR

FHL2

ACADM

APOA1

MED28

NFYB

MED13L

G0S2

NCOA3

MED20

MED1

NR1D1

SIN3B

SLC27A1

HELZ2

MED18

NR1H2

PPARG

GPS2

CDK8

MED10

ARNTL

MED6

HMGCS2

GRHL1

CLOCK

CD36

AGT

MED4

TGS1

GLIPR1

NCOA2

CYP1A1

MED9

NCOR1

CARM1

RGL1

ARNT

TNFRSF21

TIAM2

PLIN2

MED22

ANKRD1

FADS1

ACSL1

MED21

CDK19

PPARGC1B

MED7

MED19

CPT2

APOA2

MED8

MED27

ACOX1

MED11

FABP1

MED30

ABCA1

PEX11A

ANGPTL4

CYP7A1

SIN3A

NPAS2

HDAC3

ARNT2

ESRRA

MED16

CHD9

TBL1XR1

MED14

MED12

SP1

RXRA

PPARA

CYP4A11

MTF1

NCOR2

TXNRD1

NCOA6

SREBF2

RXRB

UGT1A9

CYP51A1

MTMR7

NDUFAB1

ACSM3

BZRAP1

PON1

CROT

GDE1

OSBPL7

AGK

ALDH3B1

ARSD

PNPLA4

STARD3NL

ABHD5

PIK3C2A

MBTPS2

ELOVL5

ALOX5

DPEP1

AGPS

CYP24A1

OSBPL5

AGPAT4

PNPLA6

CYP46A1

PI4K2B

INPP4A

HEXB

PIK3CB

PLEKHA5

MSMO1

GALC

TBXAS1

ACAA1

ARSF

DGAT2

SPHK2

MTMR1

CTSA

FAR2

ELOVL1

IDI1

PPAP2A

ACSL4

PLA2G10

SLC44A1

GBA2

CHAT

CSNK2A2

OSBPL3

PRKACA

ALDH3A2

ACADVL

ABCB11

PTGS2

MGLL

PTPLAD1

ACAT1

PLD1

ACACB

ACER3

PIK3C3

SYNJ2

OSBPL6

LIPE

LRP2

AACS

OXCT1

SLC27A5

STARD7

SLCO1A2

HADHA

AKR1B1

CPNE3

DDHD2

HSD17B2

ACOX3

MTMR2

HSD17B14

ACHE

PGS1

LPCAT2

CRLS1

SPTLC1

CERS4

PITPNM2

OSBPL8

PITPNM3

PTGS1

CRAT

ACOT7

SCD

HSD3B7

TECR

GGT5

GGT1

SLC25A1

PLA2G3

CYP2D6

SBF1

CHKB

MCAT

ARSA

TSPO

MTMR3

PNPLA5

PNPLA3

SLC25A17

CERK

ABHD4

DDHD1

SPTLC2

LGMN

SLC10A1

CPNE6

SEC23A

CSNK2A1

CDS2

ACOT8

VAPA

LPIN2

STS

ABCD1

BMX

MTMR8

TAZ

PCYT1B

GLA

GDPD3

VAC14

SMPD3

PLA2G15

FA2H

MLYCD

ABCC1

UBE2I

CDIPT

ACSBG1

DECR1

SQLE

MTMR9

ASAH1

LHB

PLD3

PIAS4

PRKD2

PLIN3

PLA2G4C

PLEKHA4

PIK3R2

PIK3CG

PON3

PON2

CAV1

PLEKHA8

MOGAT3

PRKAG2

PTGR1

PIP5K1B

PTGDS

PHYH

CUBN

PLEKHA1

ACBD5

MTMR4

KPNB1

HSD17B1

ALOX12

ABCC3

DHRS7B

INPP4B

SC5D

OSBP

PTPMT1

PITPNM1

CHKA

MVK

PTGES3

CYP27B1

ACSS3

LTA4H

ACAD10

VDR

CHPT1

LPCAT3

SLCO1B3

PPARD

ACOT13

FIG4

COL4A3BP

ARSB

SEC24A

EHHADH

PCCB

BCHE

SCAP

PIKFYVE

POMC

GPD2

ACADL

PECR

NEU2

PRKD3

PLEKHA3

DHCR24

SCP2

MECR

PLA2G4A

HAO2

GNPAT

PLA2G2D

HMGCL

PIK3R3

FAAH

HSD11B1

OSBPL9

ESYT2

ELOVL4

RAB14

KDSR

ACOT2

ELOVL3

GPAM

ACAT2

EPHX2

PLBD1

FABP3

PIK3CA

OCRL

AKR1D1

ACADS

ACOT9

ORMDL2

LPGAT1

PLA2G12A

ACSL3

MOGAT1

VAPB

PTGIS

MCEE

SLC10A2

MBOAT7

INSIG2

GPCPD1

SGPP1

HRASLS

PLA2G5

ECHS1

SRD5A3

ORMDL1

SUMF2

PLD2

SLC44A2

ACSBG2

CYP2E1

PNPLA7

OSBPL2

HSD17B3

PPT1

HACL1

ACLY

STARD3

PRKAB2

HSD17B7

RAN

INPP5K

ALDH3B2

LPIN3

TPTE2

ALOX5AP

PEMT

PIK3C2B

CSNK1G2

RARRES3

HRASLS2

MORC2

SBF2

HSD17B4

CEPT1

ARF3

LPIN1

SLCO1B1

CYP2J2

FADS2

PNPLA8

HILPDA

CGA

B4GALNT1

SMPD2

CYP27A1

SMPD4

BAAT

FDX1

CYP19A1

SLC44A5

EPT1

HADHB

CYP1B1

CYP2C9

CYP2C8

CH25H

AGPAT9

HADH

SEC24B

PIK3C2G

ETNK1

GLTP

MTMR6

CERS5

ESYT1

FITM1

PTGR2

SLC27A2

CYP11A1

CYP1A2

AC092724.1

MBTPS1

DPEP3

PCTP

ARSG

OSBPL1A

PIK3R5

PPAP2C

PRKACB

CYP4B1

SLC44A3

PI4KB

PIP5K1A

CERS2

SLC27A3

DEGS1

ARF1

MBOAT2

LBR

ETNK2

PLEKHA6

SUMF1

RAB5A

OSBPL10

PLA1A

SCD5

GC

FABP2

CBR4

SRD5A1

PIK3R1

TNFAIP8

MUT

HMGCLL1

CYP39A1

EBP

AWAT2

NSDHL

STAR

UGCG

PTGES2

FAM73B

PTGES

IDI2

INPP5E

CYP17A1

HSD17B12

TM7SF2

MTMR12

PIP4K2A

SEC24D

THRSP

MMAA

AKR1C2

OLAH

GPD1L

SAR1B

GGPS1

ACOXL

PLA2R1

LPCAT1

GDPD1

CERS3

CYP2U1

AGPAT5

PI4K2A

DBI

PTDSS1

SAMD8

ARSE

FAM213B

ABHD3

ESYT3

GDPD5

AGPAT6

PLA2G2F

SYNJ1

CBR1

PLA2G4D

THEM4

AGPAT3

LSS

FDPS

CYP3A4

CYP11B1

PCYT1A

BDH1

FDXR

ALOX15

NEU3

CYP4A22

ACOT11

PPAP2B

PRKAA2

ACP6

MAPKAPK2

SGPP2

HPGDS

TNFAIP8L2

ALPI

PMVK

GLB1L

PPM1L

CDS1

PTPN13

ALB

MTMR14

PLB1

LIPH

SGMS2

BDH2

ETNPPL

HPGD

ELOVL7

STARD4

ARSK

ENPP6

ACSL6

FABP7

FABP5

PRKACG

MID1IP1

SPTSSA

INPPL1

FAAH2

TMEM55B

CYP2C19

SGPL1

SMPD1

MOGAT2

PLD4

PLIN1

PIP4K2C

MTMR10

ACSF2

DPEP2

ACAA2

GPX4

MVD

GPD1

ACER1

ECI1

HRASLS5

RAB4A

ACOX2

DEGS2

MFSD2A

GSTM4

PLA2G4F

INPP5D

B3GALNT1

PLEKHA2

AGPAT2

FASN

FABP6

GLB1

FABP4

HSD17B13

ELOVL6

PLA2G1B

MTM1

PIK3CD

PTEN

CYP4F11

CYP4F22

ORMDL3

MBOAT1

CERS6

SPTLC3

STARD5

ACOT12

PPP1CA

CYP7B1

DHCR7

LCLAT1

ACSM6

ARV1

PHOSPHO1

STARD6

UGT8

PTDSS2

PCCA

GPX2

SPHK1

CIDEA

ACBD7

HSD11B2

LPCAT4

PLA2G16

ACSF3

RUFY1

SEC24C

ACER2

ACOT4

GBA

PNPLA2

SLC25A20

CPNE7

CYP11B2

ALOXE3

ALOX12B

ALOX15B

PLD6

TMEM86B

CYP8B1

FAM73A

ARSJ

PITPNB

ACBD4

ENPP7

TNFAIP8L3

ARSI

DGAT2L6

ACOT1

PRKD1

PLA2G6

DGAT1

INPP5J

TNFAIP8L1

PCYT2

CYP2R1

PIP5K1C

CYP4F2

GPAT2

PPP1CC

INSIG1

CYP4F8

CYP4F3

AKR1C1

CIDEC

AGMO

PLA2G4E

PLA2G2A

ASAH2

SUMO2

PLA2G2E

PTPLAD2

LIPI

AKR1C3

THEM5

PIK3R4

GK2

SPTSSB

GM2A

ACSL5

FITM2

SLC22A5

FABP12

FAR1

PSAP

ELOVL2

HSD17B11

AKR1C4

ECI2

OXCT2

GK

INPP5F

SGMS1

HSD3B1

HSD3B2

NEU4

AWAT1

HSD17B8

AGPAT1

SLC44A4

NEU1

CSNK2B

FABP9

CPT1B

ARSH

ACOT6

TECRL

PPAPDC2

PTPLB

SACM1L

LTC4S

HEXA

PPP1CB

NUDT19

CPNE1

STARD10

PPT2

CERS1

CPTP

AKR1B15

ACBD6

CYP21A2

GPX1

ACAD11

PISD

PI4KA

AMACR

DECR2

PLA2G4B

HTD2

FDX1L

TPTE

PIK3R6

PIP4K2B

SRD5A2

ACACA

ZNF638

KLF5

TGFB1

NFKB1

CCND3

EGR2

PCK1

WNT1

CDK4

KLF4

ADIRF

EBF1

WNT10B

CEBPB

RELA

LEP

LPL

ADIPOQ

ZNF467

SLC2A4

NR2F2

CEBPD

TNF

CEBPA

DGKZ

DGKE

DGKD

DGKH

JMJD7-PLA2G4B

PLA2G12B

LYPLA1

ADPRM

DGKQ

DGKB

DGKG

DGKA

DGKI

LYPLA2

PLA2G2C

LCAT
