# Supplementary material for: In Silico Establishment and Validation of Novel Lipid Metabolism-Related Gene Signature in Bladder Cancer
Source: Oxid Med Cell Longev. 2022 Apr 18;2022:3170950. doi: 10.1155/2022/3170950 (PMC9038413; doi:10.1155/2022/3170950)
Supplement: Supplementary Materials — Figure S1: flow diagram of the current study. Figure S2: GO analysis of coexpressed genes. GO enrichment of genes in the turquoise (A) and brown (B) modules. Figure S3: construction of the model. (A) LASSO coefficient profile plot. (B) The values of lambda in the model. Figure S4: gene mutations between the two risk subgroups. (A) Mutation analysis of the five genes. Gene mutation distributions in high-risk (B) and low-risk subgroups (C). Figure S5: correlation analysis of risk score with clinical characteristics in age (A), grade (B), clinical stage (C), M stage (D), N stage (E), and T stage (F). Figure S6: stratified survival analysis. (A) Survival analysis of two risk subgroups in age ≤ 65 years. (B) Survival analysis of two risk subgroups in age > 65. (C) Survival analysis of two risk subgroups in females. (D) Survival analysis of two risk subgroups in males. (E) Survival analysis of two risk subgroups in stages I + II. (F) Survival analysis of two risk subgroups in stages III + IV. (G) Survival analysis of two risk subgroups in T1 + T2. (H) Survival analysis of two risk subgroups in T3 + T4. (I) Survival analysis of two risk subgroups in N0. (J) Survival analysis of two risk subgroups in N1 + N2 + N3. Figure S7. The proportional hazards assumption was tested using the Schoenfeld residual test. Figure S8: correlation between the risk model and immune checkpoint inhibitors. (A) Immune-related pathways were quantified between the two risk subgroups. The relationship between risk score and PD-1 (B), PD-L1 (C), CTLA4 (D), and LAG3 (E). Figure S9: CCK-8 assay showed that silencing FASN (A, B) or TM4SF1 (C, D) suppressed cell proliferation in T24 and UMUC3 cell lines. Table S1: lipid metabolism-related genes. Table S2: clinical characteristics of 50 bladder cancer patients. Table S3: primer information. Table S4: 907 genes in turquoise module and 560 genes in brown module. Table S5: 46 genes with significant prognostic differences. [file 3170950.f1.zip › Table S4. 907 genes in turquoise module and 560 genes in brown module.doc]

**Table S4: 907 genes in turquoise module and 560 genes in brown module.**

| **turquoise module** | **brown module** |
| --- | --- |
| SPINK1 | EEF1A2 |
| KRT20 | JCHAIN |
| UPK1A | CXCL10 |
| DHRS2 | CXCL9 |
| HMGCS2 | SLITRK6 |
| UPK3A | CHI3L1 |
| PSCA | CXCL13 |
| UPK2 | TBX1 |
| CYP4B1 | CCL18 |
| SNX31 | C3 |
| UPK1B | SAA1 |
| HPGD | IGLL5 |
| SLC14A1 | C1QB |
| SLC44A4 | LYZ |
| PLA2G2F | MMP9 |
| FAM3B | MMP12 |
| CYP4F22 | C1QA |
| AKR1C2 | HLA-DRA |
| TMPRSS2 | HLA-DRB5 |
| UPK3B | SPP1 |
| AGR2 | CCL5 |
| PCP4L1 | FCGR3A |
| SCNN1B | PLAAT4 |
| BAMBI | HLA-DRB1 |
| BCAS1 | C1QC |
| PALM3 | HLA-DPA1 |
| BMP3 | HLA-DQA2 |
| SPTSSB | HLA-DQB1 |
| VSIG2 | HLA-DQA1 |
| GDF15 | APOE |
| INA | HLA-DPB1 |
| TBX2 | PSMB9 |
| MAL | TYMP |
| SCNN1G | SERPINA1 |
| SNCG | CD74 |
| KLHDC7A | CD14 |
| BTBD16 | C1R |
| ACER2 | FCER1G |
| CLDN3 | IL32 |
| FOXQ1 | RGS1 |
| GRHL3 | SECTM1 |
| FABP6 | SERPING1 |
| SCUBE2 | CD163 |
| IGF2BP2 | IFITM3 |
| TBX3 | HCLS1 |
| ERP27 | GZMB |
| CYP4F12 | SRGN |
| ZNF486 | G0S2 |
| GDPD3 | TYROBP |
| MT2A | TMEM176B |
| FBP1 | CD53 |
| MYCL | VSIG4 |
| PPP1R3C | ICAM1 |
| MT1E | CYBB |
| TM4SF1 | BMP7 |
| GSTM3 | HLA-B |
| TNNC1 | LAPTM5 |
| SLC16A1 | SEMA6A |
| IGFBP3 | IDO1 |
| REEP6 | ITGB2 |
| VTCN1 | EMP3 |
| PRR15L | GZMA |
| CAPN5 | NKG7 |
| TMEM45B | HLA-DQB2 |
| F3 | PODXL2 |
| EPHB6 | LCP1 |
| TMEM139 | FPR3 |
| FAM174B | IL2RG |
| TMEM97 | AIF1 |
| TOX3 | SLC7A4 |
| TUBB6 | ALDH1A1 |
| SOX9 | FCGR2A |
| HNF1B | CD52 |
| RNF128 | UBD |
| KCNG1 | CSF1R |
| PRR15 | SLC6A8 |
| GATA3 | HLA-DMB |
| FBLN1 | ALOX5AP |
| GGT6 | CD2 |
| ANXA1 | HLA-DOA |
| RGL3 | NCF2 |
| IGFBP6 | CPVL |
| ENTPD3 | CXCL11 |
| ANXA3 | SPI1 |
| SELENBP1 | MZB1 |
| CYP2J2 | GPR27 |
| MT1X | RNASE6 |
| RHOU | RNASE1 |
| FAM3D | ALDH2 |
| VGLL1 | CD40 |
| DEGS2 | TMEM176A |
| CAPS | LSP1 |
| ERICH5 | TREM2 |
| BNIPL | MAFB |
| GOLT1A | SELPLG |
| NPTXR | OLR1 |
| SEMA5A | FOLR2 |
| MSX2 | APOC1 |
| METTL7A | CST7 |
| GPR160 | CCL2 |
| CRACR2B | HLA-DMA |
| CTSE | CD3D |
| SNAI2 | STOM |
| APOL4 | LY96 |
| SLC38A5 | PLAUR |
| ARL14 | C3AR1 |
| HSD17B2 | CXCL2 |
| POF1B | GLIPR2 |
| PLIN4 | SOCS3 |
| S100A10 | TNFAIP3 |
| ABCC3 | CALHM6 |
| CD44 | SLAMF8 |
| TSPAN12 | CYP27A1 |
| FOSL1 | HAPLN3 |
| SLC7A5 | SIRPA |
| MT-ND6 | OR2I1P |
| NPIPB15 | CD3E |
| OSMR | PLEK |
| TLE2 | ADA2 |
| SP6 | GPR183 |
| CEBPA | MS4A4A |
| PPARG | LTB |
| ID4 | SLFN11 |
| CLEC2B | CD79A |
| SBK1 | FGL2 |
| ANXA9 | FYB1 |
| TMEM238L | CCL13 |
| GATM | MEX3A |
| ACSL5 | C2 |
| FOXA1 | HCK |
| FLRT3 | IL6 |
| ITM2C | IFITM2 |
| MSN | MPEG1 |
| MAOA | MS4A6A |
| MMRN2 | CX3CL1 |
| ZNF737 | TNFRSF18 |
| CCR7 | BIN1 |
| PPFIBP2 | IER3 |
| MT1G | FKBP5 |
| ALOX5 | PNCK |
| FBXO27 | SASH3 |
| ST3GAL5 | EVI2B |
| SORL1 | PRF1 |
| ZBTB7C | BIRC3 |
| FOXO6 | RAC2 |
| ISYNA1 | C5AR1 |
| KRT7 | CD7 |
| TNFAIP2 | MRC1 |
| PRR36 | WARS1 |
| TFPI2 | IGSF9 |
| VIPR1 | CD4 |
| PKIA | SERPINB9 |
| ST3GAL4 | IL7R |
| TESC | CYP1B1 |
| HCAR1 | BCL2A1 |
| ARHGAP40 | F13A1 |
| BTG2 | CXCR4 |
| QPCT | VAMP5 |
| MYC | IL4I1 |
| HID1 | SLC15A3 |
| FA2H | DLX5 |
| CAB39L | CCL4 |
| CNGA1 | PTPRC |
| DGAT2 | ALDOC |
| SLC16A5 | SAMHD1 |
| PDE9A | HILPDA |
| TNNT1 | MS4A7 |
| VGLL3 | THEMIS2 |
| RNF223 | TIMP1 |
| TMEM47 | P2RY6 |
| FXYD4 | GMFG |
| NDRG2 | CFI |
| GSTM4 | SLC1A3 |
| SLC9A2 | CTSL |
| NT5E | NCF4 |
| BCAT2 | SOD2 |
| KCNJ15 | APOL3 |
| LRMP | CSF1 |
| GPRC5C | CCND2 |
| CASQ1 | HAVCR2 |
| FOXJ1 | RENBP |
| IDH1 | VCAM1 |
| HSD17B14 | GPSM3 |
| MLPH | CCL3 |
| MT-ND5 | HCST |
| PITX1 | TSC22D3 |
| CD109 | ACP5 |
| FSCN1 | MSMB |
| CTSH | TNFRSF1B |
| AHNAK2 | DOK2 |
| BHLHE41 | CORO1A |
| BEX4 | CLCF1 |
| TUBB2A | CD37 |
| CYP3A5 | CTSS |
| KCNN4 | MNDA |
| RHBG | IL2RB |
| GPD1L | SLCO2B1 |
| CYB5A | FPR1 |
| DAPK1 | HLA-C |
| CGN | MAF |
| ELF5 | DERL3 |
| RADX | IL15RA |
| SLC30A2 | SOCS1 |
| MAN1C1 | PLA2G7 |
| PHLDA1 | FERMT3 |
| AK4 | GPR68 |
| TM7SF2 | CLEC7A |
| BICDL2 | PTAFR |
| FCRLB | FAS |
| KCNQ1 | ADGRE5 |
| CDH26 | GIMAP4 |
| PPM1H | MICB |
| CARD11 | FAM183A |
| HOXA13 | WIPF1 |
| C4orf19 | CCR1 |
| PRNP | SDS |
| TJP3 | PLEKHO1 |
| ADGRG6 | STING1 |
| RBBP8NL | GYPC |
| CAV2 | GBP5 |
| CHST15 | S100B |
| RAB15 | CCL4L2 |
| SRMS | COTL1 |
| GSDMB | PPP1R18 |
| KCTD15 | ATP2A3 |
| UCP2 | EVI2A |
| NXN | KCTD12 |
| TEAD4 | TLR2 |
| HS3ST6 | IL27RA |
| SLC27A2 | MCUB |
| S100A3 | CD48 |
| SCCPDH | CD86 |
| MFAP3L | C1orf162 |
| ZNF888 | INPP5D |
| GATA2 | WAS |
| ABAT | SLAMF7 |
| SMIM5 | CARD16 |
| MT-ND4L | LYN |
| SYNE4 | CD8A |
| IFI27L2 | ARHGEF19 |
| KCNS3 | GNLY |
| CLDN23 | SLC7A7 |
| RAP1GAP | CTSB |
| CLIC6 | CADM4 |
| SGK1 | FASN |
| LDHD | GIMAP7 |
| TGFBR3 | CCDC71L |
| TCEA3 | LY86 |
| DPYD | MSR1 |
| MECOM | IL10RA |
| COL9A2 | MAPK15 |
| KRT18 | HLA-G |
| LIMCH1 | SAT1 |
| LRFN4 | STAB1 |
| MYO1B | GZMH |
| EPCAM | ALDH3B1 |
| NYNRIN | SMCO4 |
| MT-ND1 | PILRA |
| ZNF626 | VSIR |
| THEM6 | VMO1 |
| PLEKHF1 | RGS10 |
| RIPPLY3 | RUNX3 |
| DDAH1 | SUSD3 |
| CROT | CXCL16 |
| GLB1L2 | CFB |
| TMC7 | LILRB4 |
| CTSC | APBB1IP |
| PLIN5 | CD300A |
| RBP1 | TCN2 |
| SLC2A6 | SRRM3 |
| FGD3 | NDRG4 |
| SPIRE2 | LIMD2 |
| CAPN13 | LAIR1 |
| ALDH4A1 | CSF2RB |
| ZNF552 | SAMSN1 |
| FSTL4 | BLVRA |
| SLC44A3 | SLC43A3 |
| TRIM47 | NRP1 |
| SLC16A3 | SLC29A2 |
| GNB4 | EPHB2 |
| PLCD1 | CSF2RA |
| FAR2 | MPP1 |
| PTPRM | TSPAN4 |
| FXYD5 | SPOCK2 |
| FGFR2 | HSD3B7 |
| LYPD6B | CYTIP |
| ID2 | LST1 |
| CD24 | ADAMDEC1 |
| CCL20 | TNFAIP8L2 |
| TOB1 | DRAM1 |
| TRAK1 | SNX10 |
| GCNT4 | ARG2 |
| IRAK2 | CCL22 |
| TMEM125 | LCP2 |
| CDA | VAV1 |
| BAIAP3 | SLA |
| RHOD | GAL3ST4 |
| NTN4 | CD27 |
| SPAG4 | CTSD |
| NIPAL1 | ST6GAL1 |
| MT-ND2 | RCOR2 |
| PLPP1 | VEGFC |
| RALGAPA2 | ARHGAP4 |
| KCNK5 | SQOR |
| SYNGR1 | UPP1 |
| SERPINB1 | OSCAR |
| TDRP | TUSC3 |
| ZNF440 | ETS1 |
| GOLGA8A | HSD11B2 |
| POU5F1 | PRXL2C |
| CDK6 | NCKAP1L |
| PLK2 | TNFSF13B |
| MT-ATP8 | EFS |
| GALNT12 | ITGAX |
| DTX4 | CD69 |
| RHPN2 | ITGAL |
| PLEKHG6 | PIK3AP1 |
| DEPTOR | TFPI |
| MT-CYB | SIGLEC1 |
| MISP3 | OSM |
| MYLIP | PLAC8 |
| CDC25B | ABI3 |
| CXADR | OPTN |
| CAPN2 | TRPV2 |
| BPGM | ACSL4 |
| KIFC2 | ITGAM |
| S100A5 | IRF8 |
| KRT8 | LAG3 |
| ERBB2 | LRRC25 |
| ACOX1 | PIK3CD |
| GALNT6 | LCK |
| ADA | CTSZ |
| EDARADD | LPXN |
| MYZAP | RAB32 |
| BSPRY | CD274 |
| PTPRR | IL2RA |
| PAQR7 | PREX1 |
| SYTL5 | HLA-DOB |
| RRAS | FAM20A |
| CDC42EP1 | LAP3 |
| ACOXL | FADS3 |
| TGFB1 | HK3 |
| LYPD6 | LITAF |
| ACSL1 | RFTN1 |
| PAQR8 | FTL |
| SLC29A3 | MARCO |
| SRCIN1 | CCL8 |
| MARCKSL1 | GIMAP2 |
| RALBP1 | TNFRSF4 |
| RASL11B | SIGLEC14 |
| HOXB5 | HLA-E |
| GSTM2 | CYTH4 |
| ALDH5A1 | RASSF4 |
| SIGLEC15 | TDRD5 |
| C6orf141 | RELB |
| FCHO1 | PARD6G |
| PLEKHA6 | GMPR |
| TSPAN6 | CTLA4 |
| STS | CD83 |
| KLHDC9 | BCL3 |
| ATP6V0E2 | JDP2 |
| SHROOM1 | EBI3 |
| ANXA2 | CIITA |
| FAAH | TNFSF12 |
| CEBPB | KIAA0040 |
| GSDME | MILR1 |
| NPAS2 | LILRB2 |
| PIM1 | FGR |
| LDHA | MFNG |
| ZNF823 | ARHGAP45 |
| SYTL2 | SLC39A8 |
| ICA1 | SIGLEC10 |
| PARVB | ARRB2 |
| BEND5 | ARHGAP30 |
| IKZF2 | GLRX |
| SH3BGRL2 | HSD11B1 |
| PIK3C2B | AOAH |
| ACSF2 | FMNL1 |
| CEMIP2 | PIM2 |
| TMEM64 | FCMR |
| ZNF662 | ADTRP |
| DNAJA4 | GPR34 |
| TSPAN15 | PDCD1LG2 |
| GPC3 | ISG20 |
| PLCE1 | TENT5C |
| ARFGEF3 | IGSF6 |
| SIDT1 | CCDC88B |
| ACSS1 | STX11 |
| GAREM1 | SPECC1 |
| ALAS1 | ARHGAP9 |
| THRB | CCR5 |
| ENGASE | SDC3 |
| B4GALT4 | SH3TC1 |
| MT-ND4 | ARID5A |
| PIGZ | TTC7A |
| MELTF | GIMAP6 |
| CCNO | TLR4 |
| SMIM6 | ADAP2 |
| UCN2 | IL6R |
| ANXA5 | SH2D2A |
| SLC2A10 | IL24 |
| ATF7IP2 | CMKLR1 |
| HSH2D | TMC8 |
| ENPP4 | SH2B3 |
| SLC45A3 | ARHGEF6 |
| MT-ATP6 | POU3F1 |
| COL27A1 | TFEB |
| CABP4 | GPR132 |
| SRC | TMSB4X |
| TLCD1 | DHCR7 |
| NR3C1 | EMILIN2 |
| SDK1 | NINJ1 |
| KLF15 | CLIC2 |
| MT-CO1 | EPB41L3 |
| CGAS | JAK3 |
| HOXB3 | BIN2 |
| SLC23A2 | FOXP3 |
| TTC39A | PLCB2 |
| CCNG2 | ZYX |
| SLC37A1 | CHI3L2 |
| PHLDB3 | NINJ2 |
| TRIM24 | PTPN7 |
| ZSCAN18 | ST6GALNAC4 |
| PABPC1L | LIPA |
| FRY | PLEKHO2 |
| UST | SIT1 |
| KIAA1211L | MYO1F |
| TPD52L1 | SEPTIN6 |
| ZNF117 | STK17B |
| C3orf14 | IL6ST |
| ZNF320 | TMEM140 |
| SLC19A2 | FTH1 |
| PARD6A | LGMN |
| BLNK | SLC9A9 |
| DENND2D | SERPINB6 |
| BMP8B | RCSD1 |
| EVX1 | DLX6 |
| MTCL1 | TPP1 |
| EPB41L1 | KCNAB2 |
| BTC | MVP |
| RHOBTB2 | CELF2 |
| PGF | PPP1R3G |
| FJX1 | ARPC1B |
| SH3GLB2 | PHLPP1 |
| PTMS | PTPRJ |
| CCNL2 | CD209 |
| KYNU | CRIP1 |
| RNF145 | ADPRH |
| NUAK2 | NFKBIE |
| SCAMP5 | NIPSNAP1 |
| CDR2L | RAB27A |
| PYROXD2 | CLEC10A |
| MCCC1 | HHEX |
| AMT | RASAL3 |
| CMYA5 | GCNT1 |
| ZNF90 | PLCL2 |
| TOP2B | AQP9 |
| PLP2 | ACTB |
| LIPE | TBXAS1 |
| ACP6 | NPC2 |
| PGM1 | LIFR |
| SOX4 | JADE2 |
| FN3K | CD8B |
| PLXNB1 | PLGRKT |
| MAPK10 | SLC11A1 |
| MKRN2OS | S1PR4 |
| RAB17 | CD247 |
| GGT7 | ACAP3 |
| ENPP5 | ACOT9 |
| SAMD10 | MAP3K14 |
| FAM107B | LAT2 |
| SC5D | FCGR1A |
| AMOT | NMT2 |
| ABCD3 | IFFO1 |
| FUCA1 | TMEM106A |
| MERTK | LILRA5 |
| ELOVL6 | KCTD14 |
| MMP15 | GIMAP8 |
| STEAP2 | CYB5R2 |
| ABHD11 | ZNF395 |
| EXT1 | PPM1M |
| PABPC1 | PAPLN |
| IRF5 | EHD1 |
| PPP1R9A | PIP4K2A |
| PATZ1 | EVL |
| RAB11FIP4 | SLC16A9 |
| TBC1D8 | PLXNC1 |
| PLEKHH1 | CD5 |
| ANKS1A | NAAA |
| CORO1C | CLEC4A |
| SIM2 | RIPPLY2 |
| ATP1A4 | MAP4K1 |
| PLCXD1 | STAT5A |
| DNMT3B | PPT1 |
| ZNF439 | JAK2 |
| PLLP | PNRC1 |
| EHD3 | HACD4 |
| MAFF | IL34 |
| CYSTM1 | RHBDF2 |
| TMEM163 | GZMM |
| SATB1 | C1orf54 |
| NADSYN1 | PSAP |
| CD59 | PALD1 |
| GALM | RNF130 |
| PPP1R37 | CCND3 |
| TIAM1 | HRCT1 |
| SMOX | TBC1D10C |
| OGFRL1 | TMEM273 |
| BDH1 | SNX8 |
| HSD17B11 | CD79B |
| PDE10A | SLC17A9 |
| STK17A | NPL |
| PFKP | NABP1 |
| MYCN | IL18BP |
| PCBD1 | ORAI1 |
| TMEM51 | CXCR3 |
| HSDL2 | GZMK |
| ZNF69 | TDO2 |
| PWWP2B | CD6 |
| ILDR1 | PARVG |
| MINDY1 | TRAF1 |
| NR2F2 | NFATC1 |
| TBC1D9 | GNG2 |
| OVGP1 | ARL11 |
| ARHGEF10L | SIRPG |
| AADAT | GSDMD |
| ITPRIP | ST8SIA4 |
| FDFT1 | NFATC2 |
| FAM13A | SYTL3 |
| B3GALNT1 | MYO1G |
| GOLGA8B | RNASE2 |
| PARD6B | CSF3R |
| CHPT1 | PDCD1 |
| ZBTB18 | TIGAR |
| PXYLP1 | RHBDL1 |
| MOCS1 | TNFAIP8 |
| SGPL1 | RIN3 |
| ZNF211 | FKBP11 |
| ARSD | SLAMF6 |
| CAPN8 | MT1H |
| BMF | TOX2 |
| ZNF43 | SOAT1 |
| CPNE8 | DOCK2 |
| CREB3L4 | VOPP1 |
| MCCC2 | NFAM1 |
| MGAT1 | C16orf54 |
| PNPLA2 | KLRB1 |
| NEIL1 | TFR2 |
| PEBP1 | RHOG |
| PM20D1 | NFKB2 |
| ZNF816 | CBR4 |
| NUDT4 | AKNA |
| ARVCF | ILVBL |
| SYT17 | RAPGEF1 |
| MCF2L | CD300LF |
| CHKA | MVK |
| FNBP1L | SLC2A5 |
| IFT140 | SEPTIN1 |
| ASAP3 | STAC3 |
| QSOX1 | ITPK1 |
| IMPDH1 |  |
| HIC2 |  |
| ZSCAN16 |  |
| ALOX15 |  |
| C20orf96 |  |
| AP1G2 |  |
| LPIN3 |  |
| CLIP4 |  |
| ASAH1 |  |
| SEC14L2 |  |
| TLE6 |  |
| ORMDL3 |  |
| IKZF3 |  |
| ZC4H2 |  |
| ATAD3C |  |
| PRKCD |  |
| TMEM131L |  |
| KANK1 |  |
| MANSC1 |  |
| ZBED2 |  |
| KIF5C |  |
| SH3YL1 |  |
| ASCC2 |  |
| PIK3R3 |  |
| TP53INP1 |  |
| DHRS13 |  |
| ABHD12 |  |
| CACNB3 |  |
| TMEM63A |  |
| TESK2 |  |
| SPTLC3 |  |
| PHYH |  |
| MAGI1 |  |
| P4HTM |  |
| FAM118A |  |
| WBP1 |  |
| LARGE1 |  |
| APOBEC3C |  |
| MSI2 |  |
| NSMF |  |
| TRPV6 |  |
| ZNF83 |  |
| EPB41L4B |  |
| OGT |  |
| SLC35F2 |  |
| PRXL2B |  |
| SMAD6 |  |
| ZNF91 |  |
| ENO1 |  |
| TMEM254 |  |
| CKAP4 |  |
| AIFM3 |  |
| DHRS11 |  |
| VSIG10 |  |
| MAPK8IP3 |  |
| DYRK1B |  |
| POGK |  |
| SUOX |  |
| STARD4 |  |
| GNL3 |  |
| PPM1L |  |
| GRAMD2B |  |
| EVA1A |  |
| ZNF711 |  |
| TNFSF15 |  |
| LDLRAD3 |  |
| PIFO |  |
| TMEM171 |  |
| DNMBP |  |
| ZMYND8 |  |
| ACAA1 |  |
| LTB4R |  |
| CSAD |  |
| RSRP1 |  |
| TRAF4 |  |
| ARHGAP27 |  |
| SEPTIN8 |  |
| ZNF738 |  |
| SLC3A2 |  |
| MID1 |  |
| RNF207 |  |
| RHOC |  |
| DMBX1 |  |
| ZNF254 |  |
| PLA2G6 |  |
| TTLL3 |  |
| ANKDD1A |  |
| ACADSB |  |
| GAPDH |  |
| ACOT11 |  |
| KLF8 |  |
| WFS1 |  |
| ACOX3 |  |
| ALKAL1 |  |
| MTHFD1L |  |
| BCKDHB |  |
| SOX13 |  |
| CLSTN3 |  |
| COLGALT1 |  |
| SYTL4 |  |
| NBEAL2 |  |
| SCAP |  |
| ECI2 |  |
| NEDD4L |  |
| ALDH1A2 |  |
| NOL4L |  |
| ZNF880 |  |
| GLCCI1 |  |
| ZNF219 |  |
| ARGLU1 |  |
| AGTRAP |  |
| C2CD2 |  |
| MBOAT7 |  |
| TNK2 |  |
| ATAT1 |  |
| YJEFN3 |  |
| RABGAP1L |  |
| DOCK3 |  |
| SLC44A2 |  |
| MTSS2 |  |
| ZNF253 |  |
| ZNF121 |  |
| C2orf15 |  |
| PAQR6 |  |
| BHMT |  |
| NSUN7 |  |
| CAMK2G |  |
| NSUN6 |  |
| HPSE |  |
| CACFD1 |  |
| SLC35E2B |  |
| PGAP3 |  |
| LENG8 |  |
| GDF7 |  |
| KIAA0895L |  |
| TMEM25 |  |
| ZFHX3 |  |
| BCL6 |  |
| GPC2 |  |
| C9orf116 |  |
| PVR |  |
| TNFSF9 |  |
| NAALADL2 |  |
| CXXC1 |  |
| PBXIP1 |  |
| IP6K2 |  |
| SLC25A29 |  |
| CDH23 |  |
| TIA1 |  |
| PRSS16 |  |
| DECR1 |  |
| ZNF682 |  |
| CDR2 |  |
| CYP4F8 |  |
| DAP |  |
| ZNF704 |  |
| MST1 |  |
| TMTC4 |  |
| CCDC14 |  |
| PNP |  |
| ANKRD10 |  |
| ZNF600 |  |
| ACVR2A |  |
| HADH |  |
| TXN |  |
| SHB |  |
| SHISA5 |  |
| SLC2A8 |  |
| ZNF761 |  |
| ZNF443 |  |
| RPH3AL |  |
| ANO8 |  |
| ZNF432 |  |
| RAB40C |  |
| SNAPC1 |  |
| MB21D2 |  |
| DDX39B |  |
| EPOR |  |
| STX3 |  |
| RNF217 |  |
| PAN2 |  |
| CRYL1 |  |
| TRIM45 |  |
| G6PD |  |
| MYO15B |  |
| HDHD3 |  |
| SLC50A1 |  |
| MZF1 |  |
| TREM1 |  |
| GFOD1 |  |
| EIF5A2 |  |
| TH |  |
| WSB1 |  |
| TMEM178B |  |
| RDM1 |  |
| LRP5 |  |
| FOXO4 |  |
| TOMM34 |  |
| ADCY6 |  |
| ZNF66 |  |
| TBC1D30 |  |
| KCNC3 |  |
| TRIM62 |  |
| MAP7D1 |  |
| SPAG1 |  |
| CPAMD8 |  |
| BBC3 |  |
| FOXD1 |  |
| SVIP |  |
| DCAKD |  |
| TRIT1 |  |
| HEXIM1 |  |
| KLHL21 |  |
| MMEL1 |  |
| ZNF681 |  |
| ZBTB12 |  |
| SPAG16 |  |
| KATNAL1 |  |
| PCGF3 |  |
| ZFP3 |  |
| REPIN1 |  |
| FBXO41 |  |
| IL9R |  |
| PANX1 |  |
| WWC1 |  |
| BANK1 |  |
| EMX2 |  |
| NKTR |  |
| WDR6 |  |
| FAM221A |  |
| MPC2 |  |
| KRTAP5-9 |  |
| HIP1R |  |
| RRAS2 |  |
| TPCN1 |  |
| AOC2 |  |
| KHDC4 |  |
| ZNF841 |  |
| PBX4 |  |
| FLOT2 |  |
| HINT1 |  |
| P4HA2 |  |
| PCMTD1 |  |
| SGSM2 |  |
| PLIN3 |  |
| HOXB4 |  |
| RPS6KA6 |  |
| NAGS |  |
| ZNF610 |  |
| SEMA4G |  |
| LPAR2 |  |
| FAM234B |  |
| CLMN |  |
| ECHDC2 |  |
| TIGD2 |  |
| INTS6L |  |
| LGALS4 |  |
| PCK2 |  |
| SLC22A3 |  |
| CBR1 |  |
| GDE1 |  |
| ZFP62 |  |
| SGK2 |  |
| CEP70 |  |
| RAB11A |  |
| ANXA4 |  |
| CASTOR3 |  |
| PAIP2B |  |
| ZNF266 |  |
| PRDX6 |  |
| ZNF528 |  |
| DEPDC7 |  |
| INPP4A |  |
| FAM219B |  |
| PGPEP1 |  |
| DUSP14 |  |
| ADH1C |  |
| ZNF44 |  |
| PYGB |  |
| MIGA2 |  |
| TCAF2 |  |
| DNM2 |  |
| SETMAR |  |
| MFSD9 |  |
| GRAMD1C |  |
| PLPP5 |  |
| SLC39A11 |  |
| CRELD1 |  |
| SUMF1 |  |
| RALGPS1 |  |
| EME2 |  |
| POMGNT2 |  |
| PXMP4 |  |
| TMCO4 |  |
| ZNF33A |  |
| GOLPH3L |  |
| STK36 |  |
| NBDY |  |
| DCBLD1 |  |
| SESN1 |  |
| CCDC84 |  |
| OSBPL7 |  |
| RERE |  |
| ZBTB10 |  |
| MAP7D3 |  |
| ZNF846 |  |
| MPP6 |  |
| HCN3 |  |
| ENTPD5 |  |
| SLC25A35 |  |
| FAM149A |  |
| RPIA |  |
| NTAN1 |  |
| CBY2 |  |
| BID |  |
| AGPAT4 |  |
| FLVCR1 |  |
| ULK3 |  |
| SAMD13 |  |
| CCNT2 |  |
| RHOQ |  |
| MYD88 |  |
| ZNF513 |  |
| ENOSF1 |  |
| ERN2 |  |
| TSPAN8 |  |
| ZNF502 |  |
| KDM6B |  |
| ING4 |  |
| INPP5A |  |
| RPS6KA4 |  |
| FARP1 |  |
| ARMCX3 |  |
| MFSD6L |  |
| GRAMD4 |  |
| EXTL3 |  |
| F5 |  |
| GDPD1 |  |
| MSANTD3 |  |
| CHD3 |  |
| TMEM250 |  |
| PEX11A |  |
| CLK1 |  |
| ZNF512 |  |
| SH2B1 |  |
| CCSER1 |  |
| PGRMC2 |  |
