# Supplementary material for: In Silico Establishment and Validation of Novel Lipid Metabolism-Related Gene Signature in Bladder Cancer
Source: Oxid Med Cell Longev. 2022 Apr 18;2022:3170950. doi: 10.1155/2022/3170950 (PMC9038413; doi:10.1155/2022/3170950)
Supplement: Supplementary Materials — Figure S1: flow diagram of the current study. Figure S2: GO analysis of coexpressed genes. GO enrichment of genes in the turquoise (A) and brown (B) modules. Figure S3: construction of the model. (A) LASSO coefficient profile plot. (B) The values of lambda in the model. Figure S4: gene mutations between the two risk subgroups. (A) Mutation analysis of the five genes. Gene mutation distributions in high-risk (B) and low-risk subgroups (C). Figure S5: correlation analysis of risk score with clinical characteristics in age (A), grade (B), clinical stage (C), M stage (D), N stage (E), and T stage (F). Figure S6: stratified survival analysis. (A) Survival analysis of two risk subgroups in age ≤ 65 years. (B) Survival analysis of two risk subgroups in age > 65. (C) Survival analysis of two risk subgroups in females. (D) Survival analysis of two risk subgroups in males. (E) Survival analysis of two risk subgroups in stages I + II. (F) Survival analysis of two risk subgroups in stages III + IV. (G) Survival analysis of two risk subgroups in T1 + T2. (H) Survival analysis of two risk subgroups in T3 + T4. (I) Survival analysis of two risk subgroups in N0. (J) Survival analysis of two risk subgroups in N1 + N2 + N3. Figure S7. The proportional hazards assumption was tested using the Schoenfeld residual test. Figure S8: correlation between the risk model and immune checkpoint inhibitors. (A) Immune-related pathways were quantified between the two risk subgroups. The relationship between risk score and PD-1 (B), PD-L1 (C), CTLA4 (D), and LAG3 (E). Figure S9: CCK-8 assay showed that silencing FASN (A, B) or TM4SF1 (C, D) suppressed cell proliferation in T24 and UMUC3 cell lines. Table S1: lipid metabolism-related genes. Table S2: clinical characteristics of 50 bladder cancer patients. Table S3: primer information. Table S4: 907 genes in turquoise module and 560 genes in brown module. Table S5: 46 genes with significant prognostic differences. [file 3170950.f1.zip › Table S5. 46 genes with significant prognostic differences.doc]

**Table S5: 46 genes with significant prognostic differences.**

FBP1

TM4SF1

EPHB6

ANXA1

CTSE

ST3GAL5

TNFAIP2

KCNJ15

MLPH

CD109

ID2

KCNK5

PTPRR

HOXB5

FAAH

NPAS2

HSH2D

HOXB3

TMEM51

IMPDH1

ZSCAN16

ZNF83

SMAD6

STARD4

LDLRAD3

CSAD

SLC3A2

TTLL3

BCL6

PVR

TIA1

ANKRD10

ZNF432

G6PD

PCGF3

HOXB4

ECHDC2

ZNF528

DUSP14

PYGB

EXTL3

CLK1

CTSS

FASN

HLA-E

FGR
